# Synthesis, crystal structure and Hirshfeld analysis of a crystalline compound comprising a 1/1 mixture of 1-[(1*R*,4*S*)- and 1-[(1*S*,4*R*)-1,7,7-trimethyl-2-oxobi­cyclo[2.2.1]heptan-3-yl­idene]hydrazinecarbo­thio­amide

**DOI:** 10.1107/S2056989019016980

**Published:** 2020-01-01

**Authors:** Fabrício Carvalho Pires, Leandro Bresolin, Vanessa Carratu Gervini, Bárbara Tirloni, Adriano Bof de Oliveira

**Affiliations:** a Escola de Química e Alimentos, Universidade Federal do Rio Grande, Av. Itália km 08, Campus Carreiros, 96203-900 Rio Grande-RS, Brazil; b Departamento de Química, Universidade Federal de Santa Maria, Av. Roraima s/n, Campus Universitário, 97105-900 Santa Maria-RS, Brazil; c Departamento de Química, Universidade Federal de Sergipe, Av. Marechal Rondon s/n, Campus Universitário, 49100-000 São Cristóvão-SE, Brazil

**Keywords:** chiral thio­semicarbazone, camphor derivative, racemic mixture, crystal structure

## Abstract

The equimolar reaction between a racemic mixture of (*R*)- and (*S*)-camphorquinone with thio­semicarbazide yielded the title compound, C_11_H_17_N_3_OS [common name: (*R*)- and (*S*)-camphor thio­semicarbazone], which maintains the chirality of the methyl­ated chiral carbon atoms and crystallizes in the centrosymmetric space group *C*2/*c*. There are two mol­ecules in general positions in the asymmetric unit, one of them being the (1*R*)-camphor thio­semicarbazone isomer and the second the (1*S*)- isomer. In the crystal, the mol­ecular units are linked by C—H⋯S, N—H⋯O and N—H⋯S inter­actions, building a tape-like structure parallel to the (01) plane, generating *R*_2_^1^(7) and *R*_2_^2^(8) graph-set motifs for the H⋯S inter­actions. The Hirshfeld surface analysis indicates that the major contributions for crystal cohesion are from H⋯H (55.00%), H⋯S (22.00%), H⋯N (8.90%) and H⋯O (8.40%) inter­actions.

## Abstract 2

A racemic mixture of (*R*)- and (*S*)-camphor thio­semicarbazone, which crystallizes in the centrosymmetric space group *C*2/*c*, is reported.

## Chemical context   

The origin of thio­semicarbazone (TSC) chemistry can be traced back to the beginning of the 20th century, when thio­semicarbazide was used for the chemical characterization of the *R*_1_ *R*_2_C=O group and it was pointed out that the *R*_1_ *R*_2_C=N—N(H)C(=S)NH_2_ compound was the main product of the condensation reaction (Freund & Schander, 1902 ▸). In the second half of the 1940′s, new insight into the TSC chemistry emerged, namely the applications in medicinal chemistry as chemotherapeutic agents against tuberculosis (Domagk *et al.*, 1946 ▸; Hoggarth *et al.*, 1949 ▸). Initially, the biological research concerning TSC derivatives was focused on the mol­ecules as free ligands, but very quickly the scope expanded to coordination compounds. One of the first reports about metal compounds of thio­semicarbazones in medicinal chemistry regards a Cu^II^ complex with *Mycobacterium tuberculosis* growth inhibition activity that was published few years later (Kuhn & Zilliken, 1954 ▸). Another milestone in this chemistry, after the reported tuberculostatic property, was the discovery of the anti­neoplastic activity of TSC derivatives in the 1960′s (Sartorelli & Booth, 1967 ▸). Concerning the mol­ecular structure of the title compound class, the N–N–C–S entity is a key feature, which has *hard* (N) and *soft* (S) donor atoms in chain (Pearson & Songstad, 1967 ▸), and so TSCs can act as *N*,*S*, *O*,*N*,*S* or *N*,*N*,*S* donors depending on the derivative.

As a result of its mol­ecular geometry, the sulfur-containing group enables the formation of several different coordination modes, including complexes with five-membered metallarings, that are well-known chelate arrangements in coordination chemistry (Lobana *et al.*, 2009 ▸). The biochemical and pharmacological applications of the TSCs is a topic that remains up-to-date and two different approaches can be considered. One is how the chemotherapeutic activity deals with the TSC compounds in form of uncoordinated ligands, so they can act as metal ion-sequestering agents for Cu^II^, Zn^II^ and Fe^II/III^ and reducing the bioavailability of these essential metals, which impacts the growth of tumor cells (Kowol *et al.*, 2016 ▸; Miklos *et al.*, 2015 ▸). The biological activity of thio­semicarbazones as metal-free mol­ecules is also possible because of the hydrogen-bonding and π–π inter­molecular inter­actions with selected biomolecules, as reported for one isatin derivative on replication inhibition of the Chikungunya virus *in silico* and *in vitro* (Mishra *et al.*, 2016 ▸). The second approach deals with the biological activity of coordination compounds, with TSCs acting as ligands. For example, Pd^II^ complexes with cinnamaldehyde-thio­semicarbazone turned out to be very active on Human Topoisomerase IIα (TOP2A) inhibition *in vitro*, a key biological target for cancer research (Rocha *et al.*, 2019 ▸), and the Au^III^ coordination compound with vaniline-thio­semi­carbazone, which has shown anti­malarial and anti­tubercular activity in *in vitro* assays (Khanye *et al.*, 2011 ▸). Thus, the synthesis and structural determination of new thio­semicarbazone derivatives is a topic of current inter­est in the field of medicinal chemistry.
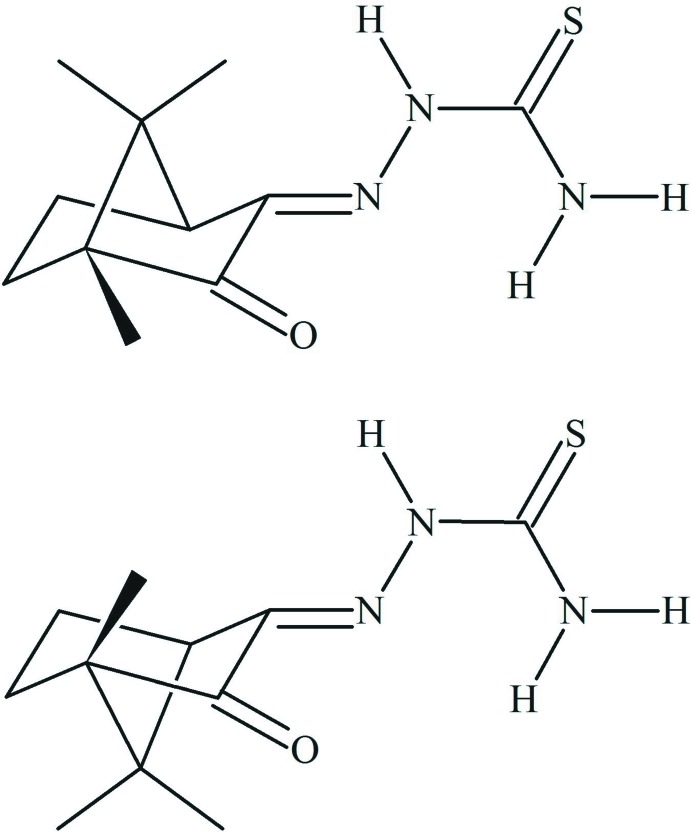


## Structural commentary   

**Figure 1:**
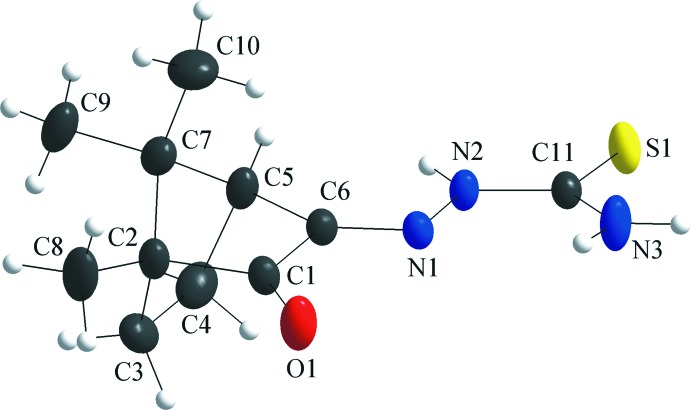
The mol­ecular structure of (1*R*)-camphor thio­semicarbazone in the asymmetric unit, showing the atom labelling and displacement ellipsoids drawn at the 40% probability level. The (1*S*)-isomer was omitted for clarity.

**Figure 2:**
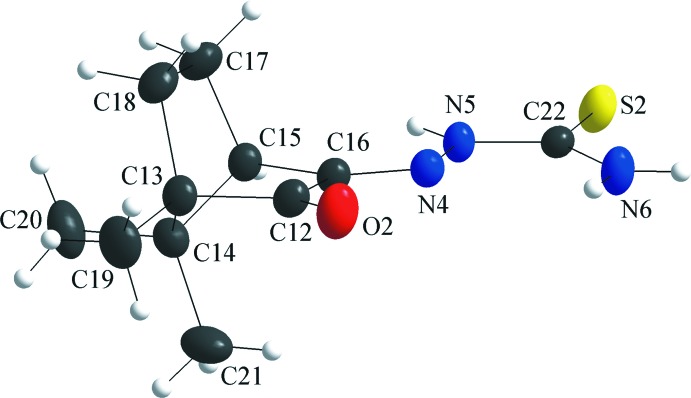
The mol­ecular structure of the second isomer of the title compound in the asymmetric unit, (1*S*)-camphor thio­semicarbazone, showing the atom labelling and displacement ellipsoids drawn at the 40% probability level. The (1*R*)-isomer was omitted for clarity.

A racemic mixture of camphorquinone was used for the synthesis of the title compound and as a result the thio­semicarbazone derivative was obtained in a 1/1 mixture of the two isomers. The asymmetric unit comprises two mol­ecules of the camphor thio­semicarbazone derivative, one of them being the (1*R*)- and the other the (1*S*)-isomer. For the first mol­ecule, the 1*R* and the 4*S* chiral centers are labelled C2 and C5, and the thio­semicarbazone unit is nearly planar with a N1—N2—C11—N3 torsion angle of −4.7 (2)° (Fig. 1 ▸). In the second mol­ecule, the 1*S* and 4*R* chiral centers are at C13 and C15, and the thio­semicarbazone fragment shows also a slight distortion from the planarity, the torsion angle for the N4—N5—C22—N6 chain being 2.4 (2)° (Fig. 2 ▸). The two mol­ecules of the asymmetric unit are shown separately for clarity and the torsion angles about the chiral C atoms are listed in Table 1 ▸.

## Supra­molecular features and Hirshfeld surface analysis   

**Figure 3:**
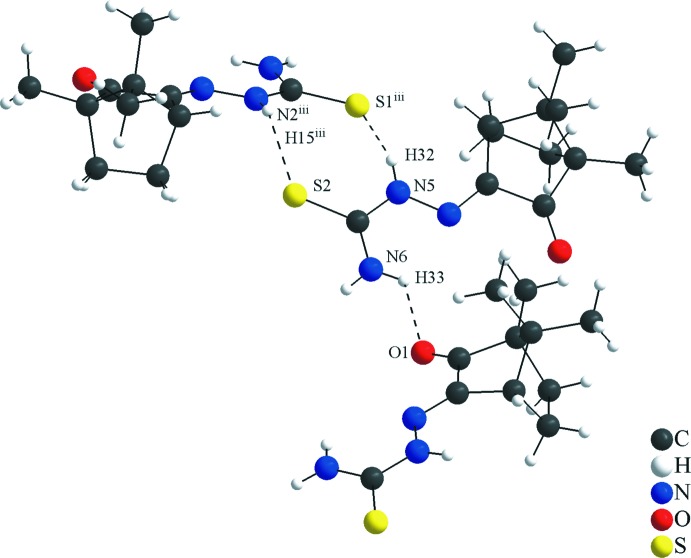
Section of the crystal structure of the title compound showing the H⋯S and H⋯O inter­molecular inter­actions for the (1*S*)-camphor thio­semicarbazone mol­ecule. The graph-set motif for the hydrogen-bonding inter­actions (dashed lines) in the crystal packing is 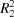
(8). The N6—H33⋯O1 inter­action connects the two mol­ecules of the asymmetric unit.

**Figure 5:**
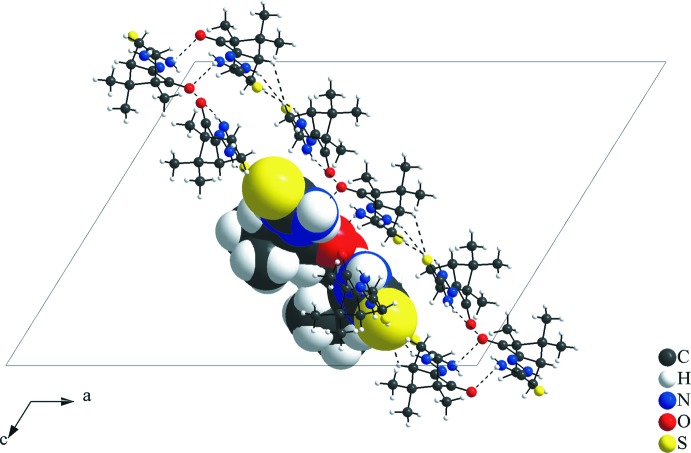
Partial crystal packing of the title compound, viewed down the [010] direction. The H⋯S and H⋯O inter­actions are shown as dashed lines and connect the mol­ecules into a tape-like structure along the (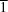
01) plane. The asymmetric unit is drawn in space-filling mode and the figure is simplified for clarity.

In the asymmetric unit, the mol­ecules in general positions are connected by the N6—H33⋯O1 inter­action. As suggested by the apolar organic periphery of the camphor fragment, the relevant and the strongest inter­molecular inter­actions are observed mainly in the thio­semicarbazone and the ketone groups. In the crystal, the mol­ecular units are linked by N2—H15⋯S2^i^, N3—H17⋯O1^ii^, C5—H5⋯S2^i^ and N5—H32⋯S1^iii^ inter­actions (Figs. 3 ▸ and 4 ▸, Table 2 ▸) into a two-dimensional hydrogen-bonded network parallel to the (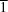
01) plane (Fig. 5 ▸). In addition, the S2–C22–N5–H32 and S1–C11–N2–H15 atom chains are subunits of the periodic arrangement, with graph-set motif 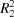
(8). Another ring-like structure is observed for the S2⋯H5–C5–C6–N1–N2–H15 atom sequence, in which the sulfur atom acts as a hydrogen-bond acceptor and bridges two *D*—H⋯S inter­actions, building an 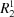
(7) motif. Since the mol­ecules crystallize in the centrosymmetric space group *C*2/*c*, chirality does not rise from the mol­ecular to the crystal structure level.

**Figure 6:**
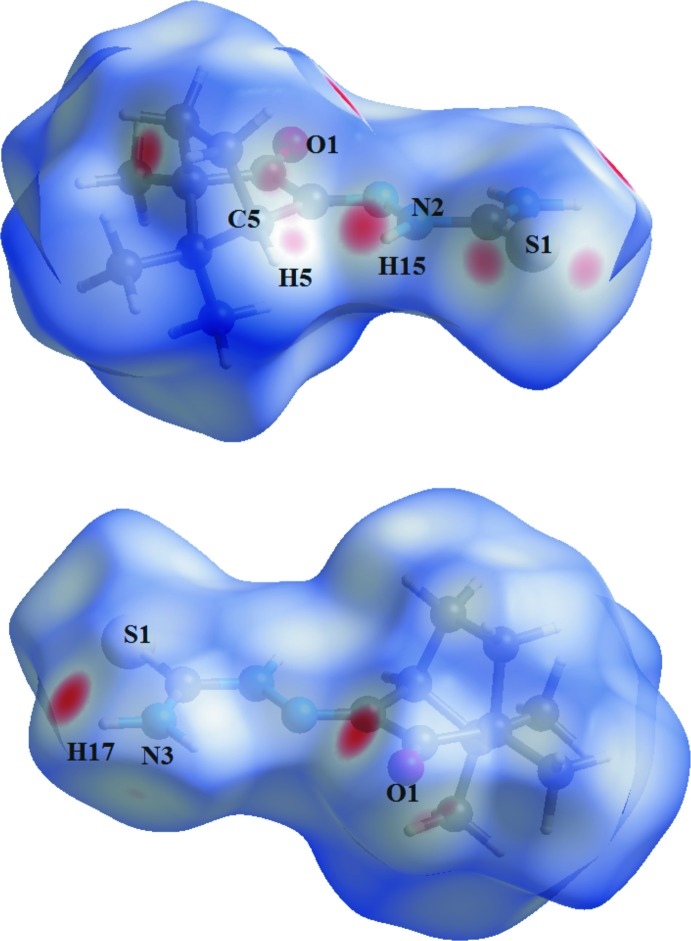
Two views of the Hirshfeld surface graphical representation (*d*_norm_) for the (1*R*)-camphor thio­semicarbazone mol­ecule. The surface is drawn with transparency and simplified for clarity. The surface regions with the strongest inter­molecular inter­actions are shown in magenta and the respective atoms are labelled. The (1*R*)- and (1*S*)-isomers are shown in separate figures for clarity [*d*_norm_ range: −0.216 to 1.411 Å].

**Figure 8:**
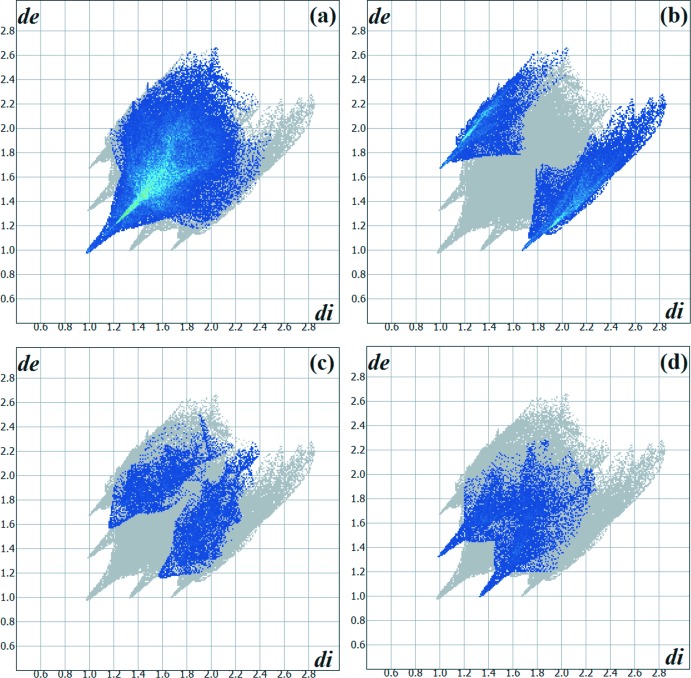
Hirshfeld surface two-dimensional fingerprint plot for the title compound showing (*a*) H⋯H, (*b*) H⋯S/S⋯H, (*c*) H⋯N/N⋯H and (*d*) H⋯O/O⋯H contacts in detail (cyan dots). The contributions of the inter­actions to the crystal packing amount to 55.0, 22.0, 8.9 and 8.4%, respectively. The *d*_e_ and *d*_i_ values are the closest external and inter­nal distances (values in Å) from given points on the Hirshfeld surface.

The Hirshfeld surface analysis (Hirshfeld, 1977 ▸) of the crystal structure suggests that the most important inter­molecular inter­actions for crystal cohesion are the following (in %): H⋯H = 50.0, H⋯S/S⋯H = 22.0, H⋯N/N⋯H = 8.9 and H⋯O/O⋯H = 8.4. For clarity, the mol­ecules in the asymmetric unit are represented using a ‘ball-and-stick’ model with transparency, in two opposite views and separate figures. The strongest inter­molecular inter­actions are located over the thio­semicarbazone and the ketone entities, as show by the graphical representation of the Hirshfeld surface for the mol­ecular units in magenta, *e.g*. the N—H, C—H, O and S atoms (Figs. 6 ▸ and 7 ▸). The contributions to the crystal packing are also shown as two-dimensional Hirshfeld surface fingerprint plots with cyan dots (Wolff *et al.*, 2012 ▸). The *d*_e_ (*y* axis) and *d*_i_ (*x* axis) values are the closest external and inter­nal distances (values in Å) from given points on the Hirshfeld surface contacts (Fig. 8 ▸).

## Database survey   

**Figure 9:**
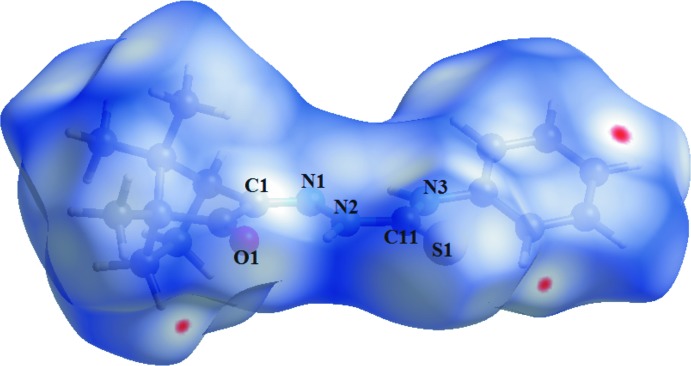
Graphical representation of the Hirshfeld surface (*d*_norm_) for the (*R*)-camphor 4-phenyl­thio­semicarbazone, the TSC derivative selected for comparison with the title compound. The surface is drawn with transparency and simplified for clarity. The surface regions with strongest inter­molecular inter­actions are shown in magenta and key atoms for the crystal packing are labelled [*d*_norm_ range: −0.003 to 1.198 Å].

**Figure 10:**
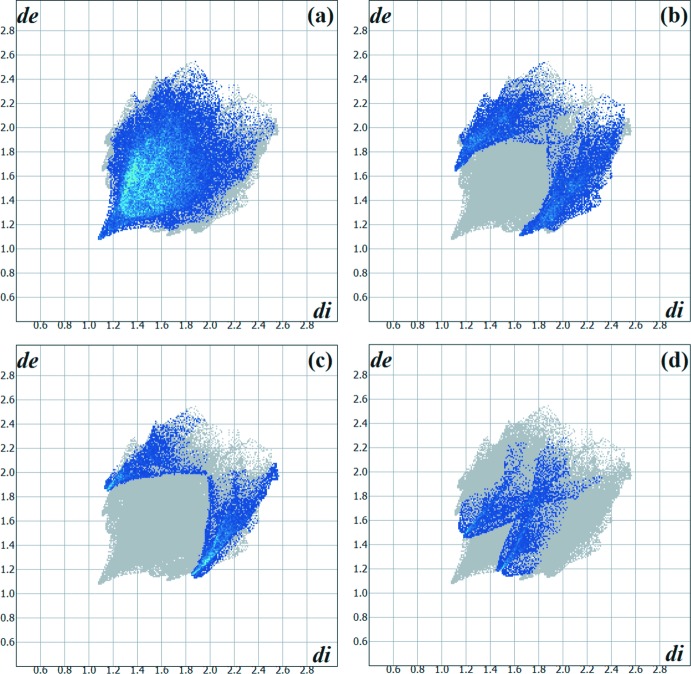
Hirshfeld surface two-dimensional fingerprint plot for the (*R*)-camphor 4-phenyl­thio­semicarbazone reference compound showing the (*a*) H⋯H, (*b*) H⋯C/C⋯H, (*c*) H⋯S/S⋯H and (*d*) H⋯N/N⋯H contacts in detail (cyan dots). The contributions of the inter­actions to the crystal packing amount to 55.9, 16.8, 11.0 and 7.8%, respectively. The *d*_e_ and *d*_i_ values are the closest external and inter­nal distances (values in Å) from given points on the Hirshfeld surface.

To the best of pur knowledge and from using database tools such as *SciFinder* (Chemical Abstracts Service, 2019 ▸), there are very few examples of thio­semicarbazone derivatives from camphorquinone. The mol­ecule selected for comparison with the title compound is (*R*)-camphor 4-phenyl­thio­semi­carbazone (Oliveira *et al.*, 2016 ▸). In both of the crystal structures, the camphor entity, with the apolar periphery and steric effect, leads to a high contribution of the H⋯H inter­molecular inter­actions for the crystal packing, being 55.00% for the title compound and 55.90% for (*R*)-camphor 4-phen­yl­thio­semicarbazone. For the literature structure, the decrease of the contributions from other possible inter­actions is assumed to be due to the geometric impediment of the phenyl ring. The impact of steric effects on the inter­molecular inter­actions sites can be seen in the graphical representation of the Hirshfeld surface in Fig. 9 ▸. In addition, the two-dimensional Hirshfeld surface fingerprint plots confirm the relationship between the mol­ecular structure and the contribution of the inter­molecular inter­actions for crystal cohesion (Fig. 10 ▸). Thus, it can be assumed that (*R*)-camphor 4-phenyl-TSC mol­ecules crystallize as discrete units, being connect by very weak inter­actions. The most frequent inter­molecular inter­actions for the crystal cohesion of the phenyl-TSC derivative are (in %) H⋯H = 55.9, H⋯C/C⋯H = 16.8, H⋯S/S⋯H = 11.0, H⋯O/O⋯H = 7.8 and H⋯N/N⋯H = 7.0. The replace­ment of one H atom by the phenyl group in the terminal amine entity strongly impacts on, for example, the contribution of the inter­molecular H⋯S/S⋯H inter­actions, which changed from 22.00% to 11.00%. Finally and remarkably, in the comparison mol­ecule, inter­molecular H⋯C/C⋯H inter­actions make the next highest contibution to the Hirshfeld surface; this inter­action is comparatively less relevant for the title compound (4.5%).

## Synthesis and crystallization   

The starting materials were commercially available and were used without further purification. The racemic mixture of *R*- and *S*-camphor was oxidized with SeO_2_ to the respective 1,2-diketone (Młochowski & Wójtowicz-Młochowska, 2015 ▸). The synthesis of the 1*R*- and 1*S*-camphor thio­semicarbazone derivative was adapted from a procedure reported previously (Freund & Schander, 1902 ▸; Oliveira *et al.* 2016 ▸). The glacial acetic acid-catalysed reaction of the 1,2-diketone (3 mmol) and thio­semicarbazide (3 mmol) in ethanol (50 ml) was refluxed funder stirring or 6 h. Single crystals suitable for X-ray diffraction were obtained from an ethanol solution by solvent evaporation. The racemic mixture of the reagent remains unchanged during the synthesis and after crystallization.

## Refinement   

Crystal data, data collection and structure refinement details are summarized in Table 3 ▸. H atoms were located in a difference-Fourier map but were positioned with idealized geometry and were refined with isotropic displacement parameters using a riding model (HFIX command) with *U*_iso_(H) = 1.2*U*_eq_(C, N) and C—H bond distances of 0.98 Å for tertiary carbon atoms and 0.97 Å for secondary C atoms. The N—H bond distances are 0.86 Å. Finally, *U*_iso_(H) = 1.5*U*_eq_(C) for the methyl groups, with C—H bond distances of 0.96 Å. A rotating model was used for the latter H atoms.

## Supplementary Material

Crystal structure: contains datablock(s) I, publication_text. DOI: 10.1107/S2056989019016980/rz5268sup1.cif

Structure factors: contains datablock(s) I. DOI: 10.1107/S2056989019016980/rz5268Isup2.hkl

CCDC reference: 1973095

Additional supporting information:  crystallographic information; 3D view; checkCIF report

## Figures and Tables

**Figure 4:**
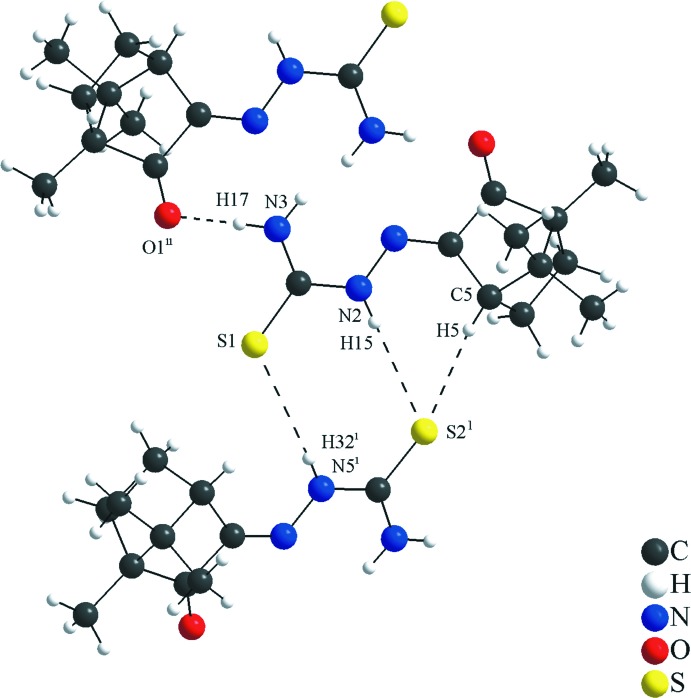
Section of the crystal structure of the title compound showing the H⋯S and H⋯O inter­molecular inter­actions for the (1*R*)-camphor thio­semicarbazone mol­ecule. H⋯S inter­actions connect the (1*R*)- and (1*S*)- isomers and the graph-set motifs for the hydrogen-bonding inter­actions (dashed lines) in the crystal packing are 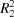
(8) and 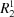 (7). The H⋯O inter­action connects two (1*R*)-isomers.

**Figure 7:**
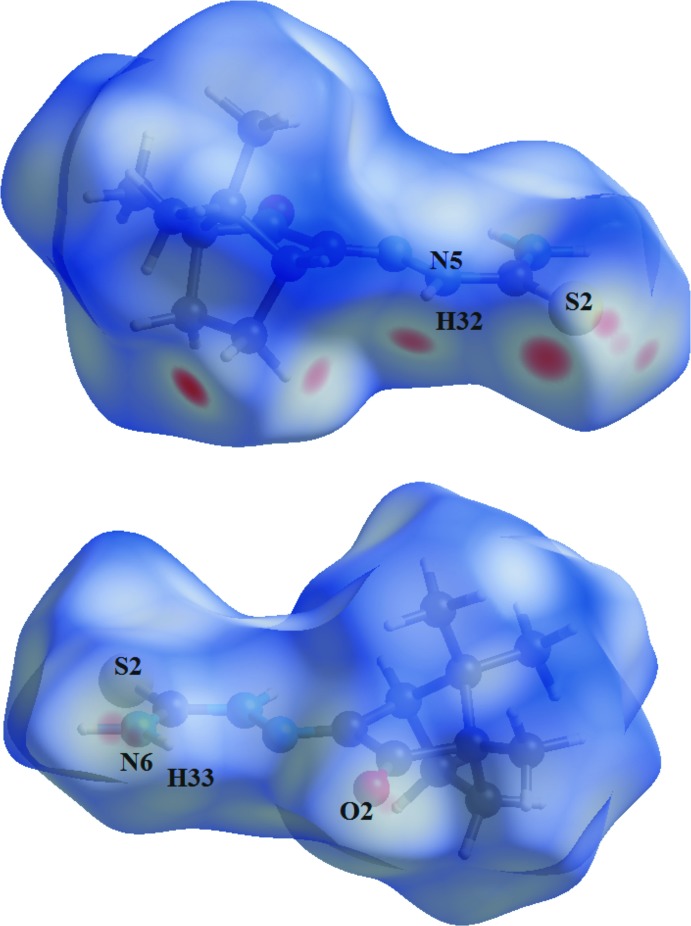
Two views of the Hirshfeld surface graphical representation (*d*_norm_) for the (1*S*)-camphor thio­semicarbazone mol­ecule. The surface is drawn with transparency and simplified for clarity. The surface regions with strongest inter­molecular inter­actions are shown in magenta and the respective atoms are labelled [*d*_norm_ range: −0.216 to 1.411 Å].

**Table 1:** Selected torsion angles (°)

Isomer	Chiral center	Atom chain	Torsion angle
*S*	C5	N1—C6—C5—C4	104.4 (2)
*S*	C5	N1—C6—C5—C7	−149.53 (17)
*R*	C2	O1—C1—C2—C3	−103.9 (2)
*R*	C2	O1—C1—C2—C7	152.42 (18)
*R*	C2	O1—C1—C2—C8	20.6 (3)
*R*	C15	N4—C16—C15—C17	−104.6 (2)
*R*	C15	N4—C16—C15—C14	148.82 (17)
*S*	C13	O2—C12—C13—C18	107.0 (2)
*S*	C13	O2—C12—C13—C14	−148.48 (18)
*S*	C13	O2—C12—C13—C19	−18.6 (3)

**Table 2:** Hydrogen-bond geometry (Å, °)

*D*—H⋯*A*	*D*—H	H⋯*A*	*D*⋯*A*	*D*—H⋯*A*
N6—H33⋯O1	0.86	2.58	2.9912 (18)	111
N2—H15⋯S2^i^	0.86	2.76	3.5413 (13)	151
N3—H17⋯O1^ii^	0.86	2.40	3.110 (2)	140
C5—H5⋯S2^i^	0.98	2.84	3.4559 (16)	122
N5—H32⋯S1^iii^	0.86	2.81	3.5334 (13)	142

Symmetry codes: (i) 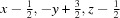; (ii) 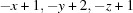; (iii) 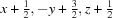.

**Table 3:** Experimental details

Crystal data	
Chemical formula	C_11_H_17_N_3_OS
*M*_r_	239.34
Crystal system, space group	Monoclinic, *C*2/*c*
Temperature (K)	296
*a*, *b*, *c* (Å)	26.6370 (9), 10.7617 (4), 20.2108 (7)
β (°)	121.932 (1)
*V* (Å^3^)	4916.9 (3)
*Z*	16
Radiation type	Cu *K*α
μ (mm^−1^)	2.21
Crystal size (mm)	0.70 × 0.46 × 0.44

Data collection	
Diffractometer	Bruker D8 Quest Photon II area detector diffractometer
Absorption correction	Multi-scan (*SADABS*; Krause *et al.*, 2015 ▸)
*T*_min_, *T*_max_	0.647, 0.754
No. of measured, independent and observed [*I* > 2σ(*I*)] reflections	47973, 4791, 4783
*R*_int_	0.026
(sin θ/λ)_max_ (Å^−1^)	0.618

Refinement	
*R*[*F* ^2^ > 2σ(*F* ^2^)], *wR*(*F* ^2^), *S*	0.043, 0.112, 1.07
No. of reflections	4791
No. of parameters	295
H-atom treatment	H-atom parameters constrained
Δρ_max_, Δρ_min_ (e Å^−3^)	0.58, −0.33

Computer programs: *APEX3* and *SAINT* (Bruker, 2015 ▸), *SHELXT2014/5* (Sheldrick, 2015*a* ▸), *SHELXL2018/3* (Sheldrick, 2015*b* ▸), *DIAMOND* (Brandenburg, 2006 ▸), *publCIF* (Westrip, 2010 ▸) and *enCIFer* (Allen *et al.*, 2004 ▸).

## supplementary crystallographic information

### Crystal data

**Table:**

C_11_H_17_N_3_OS	*F*(000) = 2048
*M**_r_* = 239.34	*D*_x_ = 1.293 Mg m^−^^3^
Monoclinic, *C*2/*c*	Cu *K*α radiation, λ = 1.54178 Å
*a* = 26.6370 (9) Å	Cell parameters from 9117 reflections
*b* = 10.7617 (4) Å	θ = 2.6–71.9°
*c* = 20.2108 (7) Å	µ = 2.21 mm^−^^1^
β = 121.932 (1)°	*T* = 296 K
*V* = 4916.9 (3) Å^3^	Block, yellow
*Z* = 16	0.70 × 0.46 × 0.44 mm

### Data collection

**Table:**

Bruker D8 Quest Photon II area detector diffractometer	4791 independent reflections
Radiation source: microfocus X ray tube, Bruker D8 Quest diffractometer	4783 reflections with *I* > 2σ(*I*)
Graphite monochromator	*R*_int_ = 0.026
φ and ω scans	θ_max_ = 72.3°, θ_min_ = 3.9°
Absorption correction: multi-scan (SADABS; Krause *et al.*, 2015)	*h* = −32→32
*T*_min_ = 0.647, *T*_max_ = 0.754	*k* = −13→13
47973 measured reflections	*l* = −24→24

### Refinement

**Table:**

Refinement on *F*^2^	Primary atom site location: structure-invariant direct methods
Least-squares matrix: full	Hydrogen site location: inferred from neighbouring sites
*R*[*F*^2^ > 2σ(*F*^2^)] = 0.043	H-atom parameters constrained
*wR*(*F*^2^) = 0.112	*w* = 1/[σ^2^(*F*_o_^2^) + (0.0548*P*)^2^ + 5.1392*P*] where *P* = (*F*_o_^2^ + 2*F*_c_^2^)/3
*S* = 1.07	(Δ/σ)_max_ = 0.001
4791 reflections	Δρ_max_ = 0.58 e Å^−^^3^
295 parameters	Δρ_min_ = −0.33 e Å^−^^3^
0 restraints	

### Special details

**Table:**

Geometry. All esds (except the esd in the dihedral angle between two l.s. planes) are estimated using the full covariance matrix. The cell esds are taken into account individually in the estimation of esds in distances, angles and torsion angles; correlations between esds in cell parameters are only used when they are defined by crystal symmetry. An approximate (isotropic) treatment of cell esds is used for estimating esds involving l.s. planes.

### Fractional atomic coordinates and isotropic or equivalent isotropic displacement parameters (Å^2^)

**Table:**

	*x*	*y*	*z*	*U*_iso_*/*U*_eq_	
C1	0.47215 (6)	0.67297 (13)	0.56684 (9)	0.0290 (3)	
C2	0.44283 (7)	0.55276 (14)	0.56677 (10)	0.0333 (3)	
C3	0.40744 (9)	0.51737 (17)	0.47745 (11)	0.0454 (4)	
H1	0.432607	0.523543	0.456262	0.054*	
H2	0.392154	0.433334	0.469767	0.054*	
C4	0.35698 (8)	0.61129 (18)	0.43863 (10)	0.0446 (4)	
H3	0.318823	0.570310	0.414527	0.054*	
H4	0.358922	0.659504	0.399497	0.054*	
C5	0.36791 (7)	0.69483 (14)	0.50817 (10)	0.0323 (3)	
H5	0.334022	0.744562	0.499147	0.039*	
C6	0.42275 (6)	0.76678 (13)	0.52963 (8)	0.0268 (3)	
C7	0.39200 (7)	0.60096 (15)	0.57601 (10)	0.0354 (4)	
C8	0.48372 (9)	0.45231 (17)	0.61983 (14)	0.0520 (5)	
H6	0.461153	0.379307	0.614501	0.078*	
H7	0.503900	0.480704	0.672855	0.078*	
H8	0.512161	0.432889	0.605930	0.078*	
C9	0.34649 (9)	0.50093 (19)	0.56284 (14)	0.0525 (5)	
H9	0.312158	0.539765	0.557730	0.079*	
H10	0.363552	0.445007	0.606443	0.079*	
H11	0.335304	0.455496	0.516133	0.079*	
C10	0.41428 (10)	0.6656 (2)	0.65375 (12)	0.0535 (5)	
H12	0.442420	0.728491	0.661405	0.080*	
H13	0.432888	0.605766	0.695165	0.080*	
H14	0.381582	0.703184	0.653700	0.080*	
C11	0.39284 (6)	1.07609 (13)	0.46828 (8)	0.0278 (3)	
N1	0.43231 (5)	0.87874 (11)	0.51846 (7)	0.0278 (3)	
N2	0.38434 (5)	0.95560 (11)	0.47974 (7)	0.0289 (3)	
H15	0.349298	0.927963	0.462906	0.035*	
N3	0.44793 (6)	1.11372 (13)	0.50042 (10)	0.0442 (4)	
H16	0.476553	1.062668	0.527236	0.053*	
H17	0.455324	1.189360	0.494626	0.053*	
O1	0.52341 (5)	0.68916 (11)	0.58743 (9)	0.0468 (3)	
S1	0.33432 (2)	1.17000 (4)	0.41603 (3)	0.04027 (14)	
C12	0.60455 (7)	0.18053 (14)	0.70270 (9)	0.0326 (3)	
C13	0.62027 (7)	0.05885 (14)	0.74694 (9)	0.0336 (3)	
C14	0.64045 (8)	0.10625 (15)	0.83037 (10)	0.0360 (4)	
C15	0.68608 (7)	0.20029 (14)	0.83429 (9)	0.0304 (3)	
H18	0.707310	0.249557	0.882240	0.037*	
C16	0.64834 (6)	0.27314 (14)	0.76081 (8)	0.0286 (3)	
C17	0.72519 (8)	0.11841 (18)	0.81668 (11)	0.0430 (4)	
H19	0.743585	0.167292	0.794814	0.052*	
H20	0.755765	0.076849	0.863374	0.052*	
C18	0.68091 (8)	0.02417 (17)	0.75681 (11)	0.0427 (4)	
H21	0.692203	−0.060168	0.775970	0.051*	
H22	0.678872	0.031572	0.707610	0.051*	
C19	0.57337 (9)	−0.04084 (18)	0.70953 (12)	0.0519 (5)	
H23	0.566827	−0.061530	0.659344	0.078*	
H24	0.537211	−0.010857	0.703438	0.078*	
H25	0.586284	−0.113455	0.742033	0.078*	
C20	0.66905 (11)	0.00551 (19)	0.89344 (12)	0.0575 (5)	
H26	0.638971	−0.049251	0.889026	0.086*	
H27	0.689268	0.043811	0.943989	0.086*	
H28	0.696813	−0.041077	0.886781	0.086*	
C21	0.59098 (10)	0.1688 (2)	0.83491 (14)	0.0557 (5)	
H29	0.572709	0.231527	0.795304	0.084*	
H30	0.607109	0.206331	0.885284	0.084*	
H31	0.561987	0.107843	0.827013	0.084*	
C22	0.69157 (6)	0.58441 (14)	0.78063 (9)	0.0283 (3)	
N4	0.64898 (6)	0.38585 (12)	0.74130 (7)	0.0304 (3)	
N5	0.69233 (6)	0.46162 (12)	0.79634 (7)	0.0304 (3)	
H32	0.719686	0.431717	0.840322	0.036*	
N6	0.64711 (6)	0.62416 (14)	0.71318 (8)	0.0415 (3)	
H33	0.620314	0.572941	0.681514	0.050*	
H34	0.644878	0.701311	0.700831	0.050*	
O2	0.56715 (6)	0.19987 (12)	0.63517 (7)	0.0497 (3)	
S2	0.74475 (2)	0.67806 (4)	0.84696 (3)	0.04220 (14)	

### Atomic displacement parameters (Å^2^)

**Table:**

	*U*^11^	*U*^22^	*U*^33^	*U*^12^	*U*^13^	*U*^23^
C1	0.0244 (7)	0.0234 (7)	0.0352 (8)	0.0009 (5)	0.0130 (6)	0.0013 (6)
C2	0.0304 (7)	0.0230 (7)	0.0484 (9)	0.0029 (6)	0.0222 (7)	0.0060 (6)
C3	0.0524 (10)	0.0368 (9)	0.0563 (11)	−0.0113 (8)	0.0351 (9)	−0.0143 (8)
C4	0.0413 (9)	0.0499 (11)	0.0352 (9)	−0.0157 (8)	0.0152 (7)	−0.0049 (8)
C5	0.0237 (7)	0.0280 (7)	0.0427 (9)	0.0014 (6)	0.0159 (6)	0.0078 (6)
C6	0.0234 (7)	0.0237 (7)	0.0299 (7)	0.0000 (5)	0.0117 (6)	0.0014 (5)
C7	0.0363 (8)	0.0321 (8)	0.0417 (9)	0.0052 (7)	0.0233 (7)	0.0085 (7)
C8	0.0439 (10)	0.0310 (9)	0.0818 (14)	0.0130 (8)	0.0337 (10)	0.0203 (9)
C9	0.0470 (10)	0.0422 (10)	0.0816 (14)	0.0027 (8)	0.0430 (11)	0.0195 (10)
C10	0.0655 (13)	0.0601 (12)	0.0417 (10)	0.0051 (10)	0.0330 (10)	0.0005 (9)
C11	0.0277 (7)	0.0222 (7)	0.0305 (7)	−0.0009 (5)	0.0134 (6)	0.0013 (5)
N1	0.0239 (6)	0.0226 (6)	0.0330 (6)	0.0014 (5)	0.0125 (5)	0.0023 (5)
N2	0.0216 (6)	0.0226 (6)	0.0379 (7)	0.0001 (5)	0.0126 (5)	0.0055 (5)
N3	0.0270 (7)	0.0284 (7)	0.0634 (10)	−0.0042 (5)	0.0144 (7)	0.0093 (6)
O1	0.0240 (6)	0.0349 (6)	0.0721 (9)	0.0016 (5)	0.0191 (6)	0.0102 (6)
S1	0.0298 (2)	0.0244 (2)	0.0521 (3)	0.00304 (14)	0.01176 (19)	0.00846 (16)
C12	0.0287 (8)	0.0290 (8)	0.0332 (8)	−0.0022 (6)	0.0117 (7)	−0.0041 (6)
C13	0.0372 (8)	0.0249 (7)	0.0352 (8)	−0.0038 (6)	0.0169 (7)	−0.0043 (6)
C14	0.0436 (9)	0.0301 (8)	0.0381 (8)	−0.0052 (7)	0.0242 (7)	−0.0030 (6)
C15	0.0300 (7)	0.0276 (7)	0.0279 (7)	−0.0014 (6)	0.0114 (6)	−0.0008 (6)
C16	0.0265 (7)	0.0261 (7)	0.0286 (7)	−0.0012 (6)	0.0114 (6)	−0.0020 (6)
C17	0.0313 (8)	0.0442 (10)	0.0486 (10)	0.0079 (7)	0.0179 (8)	0.0005 (8)
C18	0.0485 (10)	0.0343 (9)	0.0485 (10)	0.0074 (7)	0.0278 (9)	−0.0030 (7)
C19	0.0571 (12)	0.0353 (9)	0.0558 (11)	−0.0174 (9)	0.0248 (10)	−0.0104 (8)
C20	0.0831 (15)	0.0427 (11)	0.0451 (10)	−0.0081 (10)	0.0329 (11)	0.0068 (9)
C21	0.0590 (13)	0.0559 (12)	0.0738 (14)	−0.0085 (10)	0.0499 (12)	−0.0129 (10)
C22	0.0261 (7)	0.0262 (7)	0.0328 (7)	0.0011 (6)	0.0157 (6)	−0.0003 (6)
N4	0.0289 (6)	0.0268 (6)	0.0294 (6)	−0.0026 (5)	0.0113 (5)	−0.0020 (5)
N5	0.0290 (6)	0.0243 (6)	0.0284 (6)	−0.0027 (5)	0.0087 (5)	0.0000 (5)
N6	0.0361 (7)	0.0310 (7)	0.0390 (8)	−0.0008 (6)	0.0073 (6)	0.0067 (6)
O2	0.0445 (7)	0.0422 (7)	0.0343 (6)	−0.0051 (6)	0.0016 (5)	−0.0020 (5)
S2	0.0339 (2)	0.0266 (2)	0.0470 (3)	−0.00303 (15)	0.00842 (19)	−0.00593 (16)

### Geometric parameters (Å, º)

**Table:**

C1—O1	1.2111 (19)	C12—O2	1.208 (2)
C1—C6	1.506 (2)	C12—C16	1.511 (2)
C1—C2	1.511 (2)	C12—C13	1.514 (2)
C2—C8	1.506 (2)	C13—C19	1.511 (2)
C2—C7	1.550 (2)	C13—C14	1.560 (2)
C2—C3	1.579 (2)	C13—C18	1.564 (2)
C3—C4	1.526 (3)	C14—C21	1.525 (3)
C3—H1	0.9700	C14—C20	1.534 (3)
C3—H2	0.9700	C14—C15	1.551 (2)
C4—C5	1.561 (2)	C15—C16	1.500 (2)
C4—H3	0.9700	C15—C17	1.543 (2)
C4—H4	0.9700	C15—H18	0.9800
C5—C6	1.500 (2)	C16—N4	1.278 (2)
C5—C7	1.543 (2)	C17—C18	1.541 (3)
C5—H5	0.9800	C17—H19	0.9700
C6—N1	1.2760 (19)	C17—H20	0.9700
C7—C10	1.523 (3)	C18—H21	0.9700
C7—C9	1.536 (2)	C18—H22	0.9700
C8—H6	0.9600	C19—H23	0.9600
C8—H7	0.9600	C19—H24	0.9600
C8—H8	0.9600	C19—H25	0.9600
C9—H9	0.9600	C20—H26	0.9600
C9—H10	0.9600	C20—H27	0.9600
C9—H11	0.9600	C20—H28	0.9600
C10—H12	0.9600	C21—H29	0.9600
C10—H13	0.9600	C21—H30	0.9600
C10—H14	0.9600	C21—H31	0.9600
C11—N3	1.316 (2)	C22—N6	1.318 (2)
C11—N2	1.3571 (19)	C22—N5	1.3567 (19)
C11—S1	1.6810 (15)	C22—S2	1.6764 (15)
N1—N2	1.3680 (17)	N4—N5	1.3700 (17)
N2—H15	0.8600	N5—H32	0.8600
N3—H16	0.8600	N6—H33	0.8600
N3—H17	0.8600	N6—H34	0.8600
			
O1—C1—C6	127.11 (14)	O2—C12—C16	126.90 (15)
O1—C1—C2	127.73 (14)	O2—C12—C13	128.39 (14)
C6—C1—C2	105.00 (12)	C16—C12—C13	104.64 (12)
C8—C2—C1	115.78 (14)	C19—C13—C12	114.91 (14)
C8—C2—C7	120.20 (14)	C19—C13—C14	119.60 (15)
C1—C2—C7	101.38 (12)	C12—C13—C14	100.66 (12)
C8—C2—C3	114.23 (15)	C19—C13—C18	114.77 (15)
C1—C2—C3	101.74 (13)	C12—C13—C18	103.07 (13)
C7—C2—C3	100.78 (13)	C14—C13—C18	101.41 (13)
C4—C3—C2	105.01 (13)	C21—C14—C20	109.08 (16)
C4—C3—H1	110.7	C21—C14—C15	112.81 (14)
C2—C3—H1	110.7	C20—C14—C15	112.79 (15)
C4—C3—H2	110.7	C21—C14—C13	113.24 (15)
C2—C3—H2	110.7	C20—C14—C13	113.76 (14)
H1—C3—H2	108.8	C15—C14—C13	94.66 (12)
C3—C4—C5	102.97 (14)	C16—C15—C17	104.52 (13)
C3—C4—H3	111.2	C16—C15—C14	101.17 (12)
C5—C4—H3	111.2	C17—C15—C14	102.83 (13)
C3—C4—H4	111.2	C16—C15—H18	115.5
C5—C4—H4	111.2	C17—C15—H18	115.5
H3—C4—H4	109.1	C14—C15—H18	115.5
C6—C5—C7	101.38 (12)	N4—C16—C15	133.61 (14)
C6—C5—C4	104.30 (13)	N4—C16—C12	121.12 (13)
C7—C5—C4	102.40 (13)	C15—C16—C12	105.20 (12)
C6—C5—H5	115.6	C18—C17—C15	103.13 (13)
C7—C5—H5	115.6	C18—C17—H19	111.1
C4—C5—H5	115.6	C15—C17—H19	111.1
N1—C6—C5	133.70 (13)	C18—C17—H20	111.1
N1—C6—C1	121.19 (13)	C15—C17—H20	111.1
C5—C6—C1	104.97 (12)	H19—C17—H20	109.1
C10—C7—C9	109.87 (16)	C17—C18—C13	104.66 (13)
C10—C7—C5	111.71 (15)	C17—C18—H21	110.8
C9—C7—C5	112.69 (14)	C13—C18—H21	110.8
C10—C7—C2	112.86 (15)	C17—C18—H22	110.8
C9—C7—C2	113.84 (14)	C13—C18—H22	110.8
C5—C7—C2	95.24 (12)	H21—C18—H22	108.9
C2—C8—H6	109.5	C13—C19—H23	109.5
C2—C8—H7	109.5	C13—C19—H24	109.5
H6—C8—H7	109.5	H23—C19—H24	109.5
C2—C8—H8	109.5	C13—C19—H25	109.5
H6—C8—H8	109.5	H23—C19—H25	109.5
H7—C8—H8	109.5	H24—C19—H25	109.5
C7—C9—H9	109.5	C14—C20—H26	109.5
C7—C9—H10	109.5	C14—C20—H27	109.5
H9—C9—H10	109.5	H26—C20—H27	109.5
C7—C9—H11	109.5	C14—C20—H28	109.5
H9—C9—H11	109.5	H26—C20—H28	109.5
H10—C9—H11	109.5	H27—C20—H28	109.5
C7—C10—H12	109.5	C14—C21—H29	109.5
C7—C10—H13	109.5	C14—C21—H30	109.5
H12—C10—H13	109.5	H29—C21—H30	109.5
C7—C10—H14	109.5	C14—C21—H31	109.5
H12—C10—H14	109.5	H29—C21—H31	109.5
H13—C10—H14	109.5	H30—C21—H31	109.5
N3—C11—N2	116.94 (13)	N6—C22—N5	116.89 (14)
N3—C11—S1	123.15 (12)	N6—C22—S2	123.31 (12)
N2—C11—S1	119.91 (11)	N5—C22—S2	119.78 (11)
C6—N1—N2	117.31 (12)	C16—N4—N5	117.22 (12)
C11—N2—N1	119.05 (12)	C22—N5—N4	119.21 (12)
C11—N2—H15	120.5	C22—N5—H32	120.4
N1—N2—H15	120.5	N4—N5—H32	120.4
C11—N3—H16	120.0	C22—N6—H33	120.0
C11—N3—H17	120.0	C22—N6—H34	120.0
H16—N3—H17	120.0	H33—N6—H34	120.0
			
O1—C1—C2—C8	20.6 (3)	O2—C12—C13—C19	−18.6 (3)
C6—C1—C2—C8	−163.87 (15)	C16—C12—C13—C19	164.22 (15)
O1—C1—C2—C7	152.42 (18)	O2—C12—C13—C14	−148.48 (18)
C6—C1—C2—C7	−32.03 (15)	C16—C12—C13—C14	34.31 (15)
O1—C1—C2—C3	−103.9 (2)	O2—C12—C13—C18	107.0 (2)
C6—C1—C2—C3	71.67 (14)	C16—C12—C13—C18	−70.19 (15)
C8—C2—C3—C4	163.31 (14)	C19—C13—C14—C21	−62.9 (2)
C1—C2—C3—C4	−71.19 (15)	C12—C13—C14—C21	63.92 (17)
C7—C2—C3—C4	32.98 (16)	C18—C13—C14—C21	169.75 (15)
C2—C3—C4—C5	1.05 (17)	C19—C13—C14—C20	62.4 (2)
C3—C4—C5—C6	70.16 (15)	C12—C13—C14—C20	−170.78 (15)
C3—C4—C5—C7	−35.17 (16)	C18—C13—C14—C20	−64.95 (18)
C7—C5—C6—N1	−149.53 (17)	C19—C13—C14—C15	179.78 (15)
C4—C5—C6—N1	104.4 (2)	C12—C13—C14—C15	−53.36 (14)
C7—C5—C6—C1	34.96 (15)	C18—C13—C14—C15	52.47 (14)
C4—C5—C6—C1	−71.15 (15)	C21—C14—C15—C16	−64.17 (17)
O1—C1—C6—N1	−2.3 (3)	C20—C14—C15—C16	171.67 (14)
C2—C1—C6—N1	−177.88 (14)	C13—C14—C15—C16	53.46 (14)
O1—C1—C6—C5	173.92 (17)	C21—C14—C15—C17	−172.06 (15)
C2—C1—C6—C5	−1.68 (16)	C20—C14—C15—C17	63.79 (18)
C6—C5—C7—C10	64.28 (17)	C13—C14—C15—C17	−54.42 (14)
C4—C5—C7—C10	171.87 (15)	C17—C15—C16—N4	−104.6 (2)
C6—C5—C7—C9	−171.46 (14)	C14—C15—C16—N4	148.82 (17)
C4—C5—C7—C9	−63.87 (17)	C17—C15—C16—C12	72.31 (15)
C6—C5—C7—C2	−52.88 (14)	C14—C15—C16—C12	−34.24 (15)
C4—C5—C7—C2	54.71 (14)	O2—C12—C16—N4	−0.1 (3)
C8—C2—C7—C10	64.5 (2)	C13—C12—C16—N4	177.19 (14)
C1—C2—C7—C10	−64.58 (17)	O2—C12—C16—C15	−177.49 (17)
C3—C2—C7—C10	−169.03 (14)	C13—C12—C16—C15	−0.22 (16)
C8—C2—C7—C9	−61.6 (2)	C16—C15—C17—C18	−69.89 (16)
C1—C2—C7—C9	169.29 (15)	C14—C15—C17—C18	35.43 (17)
C3—C2—C7—C9	64.84 (17)	C15—C17—C18—C13	−1.30 (18)
C8—C2—C7—C5	−179.27 (16)	C19—C13—C18—C17	−163.20 (16)
C1—C2—C7—C5	51.64 (14)	C12—C13—C18—C17	71.12 (16)
C3—C2—C7—C5	−52.81 (14)	C14—C13—C18—C17	−32.81 (17)
C5—C6—N1—N2	0.9 (3)	C15—C16—N4—N5	0.2 (3)
C1—C6—N1—N2	175.84 (13)	C12—C16—N4—N5	−176.32 (13)
N3—C11—N2—N1	−4.7 (2)	N6—C22—N5—N4	2.4 (2)
S1—C11—N2—N1	176.18 (10)	S2—C22—N5—N4	−179.29 (11)
C6—N1—N2—C11	178.54 (14)	C16—N4—N5—C22	−174.20 (14)

### Hydrogen-bond geometry (Å, º)

**Table:**

*D*—H···*A*	*D*—H	H···*A*	*D*···*A*	*D*—H···*A*
N6—H33···O1	0.86	2.58	2.9912 (18)	111
N2—H15···S2^i^	0.86	2.76	3.5413 (13)	151
N3—H17···O1^ii^	0.86	2.40	3.110 (2)	140
C5—H5···S2^i^	0.98	2.84	3.4559 (16)	122
N5—H32···S1^iii^	0.86	2.81	3.5334 (13)	142

Symmetry codes: (i) *x*−1/2, −*y*+3/2, *z*−1/2; (ii) −*x*+1, −*y*+2, −*z*+1; (iii) *x*+1/2, −*y*+3/2, *z*+1/2.
